# Red-teaming as an imperative for strengthening synthetic nucleic acid screening

**DOI:** 10.3389/fbioe.2026.1841668

**Published:** 2026-06-24

**Authors:** Yousuf Khan, Ketan Thorat

**Affiliations:** Institute for Stem Cell Science and Regenerative Medicine, Bengaluru, India

**Keywords:** adversarial testing, biosecurity, nucleic acid synthesis screening, red teaming, synthetic nucleic acids, AIxBio

## Abstract

Synthetic Nucleic Acid Technologies (SNAT) have provided unprecedented precision to engineer life and are poised to transform small-molecule manufacturing, the circular economy, health security, and food security. Yet the rapid fall in synthesis costs, the convergence with AI, and the growing democratisation of SNAT have raised significant biosecurity concerns, particularly regarding the potential misuse of this technology for nefarious purposes. As a result, the global biosecurity discourse has increasingly emphasised the need for robust oversight mechanisms to ensure that SNAT is not exploited to create biological agents with large-scale destructive potential. Current oversight mechanisms rely on sequence-of-concern (SoC) screening, customer vetting, and voluntary compliance frameworks. However, these strategies have documented limitations, including static SoC databases, bypass pathways, and gaps that widen as SNAT capabilities become more distributed and adaptable. The literature contains multiple examples of how existing safeguards can miss dangerous constructs. This makes it imperative to identify shortcomings in extant screening tools and rethink SNAT regulatory strategies toward more proactive and dynamic approaches. Red-teaming, widely used in military planning, cybersecurity, and risk management, employs controlled adversarial testing to identify vulnerabilities before they can be exploited. There has been little effort to consolidate or assess the broader potential of red-teaming in the context of SNAT biosecurity. This article will examine current red-teaming approaches relevant to SNAT biosecurity, analyse how these methods have been applied in adjacent fields, and outline how similar principles could support more adaptive and dynamic screening frameworks.

## 1 Introduction

Synthetic Nucleic Acid Technology (SNAT) has transformed molecular biology into an engineerable discipline. Synthetic nucleic acids underpin technologies such as CRISPR-based gene editing, powering breakthroughs in precision therapeutics, climate-resilient agriculture, biomanufacturing and other areas. SNAT is a foundational driver of the global bioeconomy.

The first biosecurity warning bells came when different research groups began to reconstruct pathogens. The polio virus was *de novo* synthesised (Cello et al., 2002) followed by the reconstruction of the 1918 influenza virus (Tumpey et al., 2005). Today, SNAT biosecurity efforts are guided by frameworks such as the US HHS Screening Framework Guidance for Providers of Synthetic Double-Stranded DNA (Hoffmann et al., 2023) and the International Gene Synthesis Consortium (IGSC) Harmonised Screening Protocol. DNA synthesis providers serve as the primary gatekeepers, focusing on pre-synthesis screening and customer vetting (Rose et al., 2024). However, most experts recognise that present SNAT biosecurity systems are not well matched to current and predicted synthetic biology capabilities (Trump et al., 2020).

Current SNAT Regulation is largely based on identification and static classification of hazardous agents and their genetic sequences. Frameworks suffer from *inter alia* overreliance on known threats from listed sequences of concerns (SoC) (Kane and Parker, 2024), uneven enforcement, voluntary compliance. Advances such as benchtop DNA synthesisers and convergence of AI and synthetic biology have further challenged SNAT governance. Historically, corrective steps in synthetic biology and biotechnology regulation have been formulated post facto (Berger et al., 2018). Lack of anticipation leaves an alarming window where regulations are inadequate or misaligned to prevent misuse.

Red-teaming or adversarial testing can address this reactionary attitude and support anticipatory governance. Commonly used in military strategy and cybersecurity, red-teaming systematically evaluates gaps and weaknesses in a system through adversarial testing. By identifying existing blind spots or flagging potential ones, this controlled adversarial exercise helps identify future challenges. In comparison, red-teaming is rarely explored in biological sciences. Some well-known cases include Bioeconomy Information Sharing and Analysis Center (BIO-ISAC) that focuses on building cyberbiosecurity resilience through collaboration, intelligence sharing, and the development of practical security tools (ISAC, 2025); and the Biohacking Village, a non-profit organisation that employs red teaming strategies across its Device Lab, Virtual Hospital, and CTF events to identify and address cyber-physical vulnerabilities in medical devices and healthcare infrastructure (DEFCON, 2025). Red-teaming has potential applications for multiple biosecurity concerns (Zhang and Gronvall, 2020) and experts have explicitly called for incorporating red-teaming into biosecurity practices for SNAT (Koblentz, 2017; Diggans and Leproust, 2019). Herein, we discuss red-teaming as a crucial complementary layer that can augment the SNAT biosecurity discourse from a static, reactionary endeavour to a proactive, anticipatory exercise.

## 2 SNAT biosecurity landscape today

### 2.1 Screening constraints

The central tenet in SNAT biosecurity is that biological threats enabled by nucleic acid synthesis products can be mitigated by pre-synthesis screening. Commercial companies evaluate ordered nucleotide sequences against SoC databases and subject flagged orders to greater scrutiny.

Most similarity analysis is carried out using homology-based screening tools such as BLAST. However these tools were not designed to evaluate the biosecurity implications and have been shown to either miss hazardous sequences or show up false positives when applied in this context (Beal et al., 2023). The ‘black list’ approach fails to detect new threats, particularly those engineered specifically to mask any hazards (Appleton and Millett, 2021). Further, most control lists categorise risk as defined by the host organism and cannot account for unlisted organisms modified to produce hazardous traits and products (Millett et al., 2023; Gemler et al., 2024; Kane and Parker, 2024).

Screening based on sequence similarity also misses out on context and function. Parts of a target sequence, benign in isolation, may be ordered from separate vendors and then recombined to create a hazardous product. With AI, sequences can now be designed to escape current SNAT screening yet encode hazardous products (Carter et al., 2023; Wittmann et al. 2025). Therefore, systemic risks cannot be addressed by sequence-level surveillance alone. By design, biosecurity outlook in SNAT is skewed towards known threats, rather than what is biologically possible.

### 2.2 System-level constraints

Current oversight mechanisms are based on decentralised voluntary structures. IGSC and IBBIS Common Mechanism have tried to create common, interoperable international standards. However, they lack enforcement mechanisms causing patchy implementation (Diggans and Leproust, 2019; Wheeler et al., 2024). While US has incentivised synthesis screening frameworks; the Europe is still building legislative interventions such as the proposed EU Biotech bill (Diggans and Labeikovsky, 2025). However, the implementation of screening frameworks is not uniform internationally and differs across regions and companies (DiEuliis et al., 2017; Rose and Nelson, 2023; Crawford et al., 2024; Kane and Parker, 2024). International governance challenges leave loopholes whereby potential bad actors can circumvent stricter screening regimes by opting for vendors based in countries with weaker enforcement (Cameron et al., 2020; Rose et al., 2024).

The 2010 guidance (Hoffmann et al., 2023) focuses on customer vetting based on self-reported information of user identity, institutional affiliation and declared intent. However, Dual Use Research of Concern (DURC) is frequently conducted by legitimate scientists, and the frequency of DURC-relevant projects is on the rise (Selgelid, 2009; Wintle et al., 2017). Even when actors are non-malicious, synthesis vendors often lack robust in-house mechanisms to verify self-reported credentials, relying instead on time-consuming manual processes to establish customer legitimacy. Dependence on human evaluation increases likelihood of subjectivity and error (Alexanian and Carter, 2024). Further, there is no effective oversight over risks such as insider threats, reuse/repurposing of ordered sequences or secondary transfer.

### 2.3 Adaptive pressure from technology convergence

The integration of AI has democratized knowledge required, for instance, to camouflage sequences of concern and use split-and-mix techniques. AI tools can greatly enhance design capabilities and shorten design-application cycles (National Academies of Sciences et al., 2025; Kim et al., 2026). While some studies report that LLMs can lower knowledge barriers and facilitate access to information about biological threats and DURC (Service, 2023; Ramakrishnan, 2024), others have suggested that there is no significant enablement (Hong et al., 2026). Simultaneously, cases of emerging cyberbiosecurity risks such as DNA-encoded malware prove that it is crucial to consider interconnected technical domains in biosecurity strategies for SNAT (Ney et al., 2017).

Overall, the static, known-threat model that SNAT biosecurity is based on must evolve to respond to dynamic adversarial intent. MIT biosecurity expert Kevin Esvelt’s team conducted–an experiment with Federal Bureau of Investigation’s (FBI) awareness, where the researchers ordered DNA sequences for ricin toxin and 1918 influenza virus, applying various techniques to evade screening. The team succeeded in receiving the ordered sequences from the majority of vendors within and outside the USA, including IGSC members (Field, 2024; Edison et al., 2026). The IGSC responded, stating that the system worked as intended but the case highlights underlying anxieties about present-day SNAT biosecurity (International Gene Synthesis Consortium IGSC, 2024).

## 3 Red-teaming approaches

Red-teaming is a systematic, expert-driven, controlled evaluation of a system, process or policy to improve resilience. This exercise is carried out in a controlled environment to provide valuable information about blind spots, anticipation flaws, and response capacity (Longbine, 2008; Boyens et al., 2012; Zhang and Gronvall, 2020).

Red-teaming has seen extensive application in cybersecurity (Teichmann and Boticiu, 2023). Cyber systems are complex, involving multiple components, distributed networks and diverse stakeholders. Stress-testing these systems by introducing perturbations and adversarial pressure can highlight emergent vulnerabilities and blind spots missed by static or compliance-driven security measures. This includes testing on multiple layers, including technical, procedural and human (Rehberger, 2020; HICP, 2023; Roopa et al., 2025).

In biological sciences, existing red-teaming efforts can be broadly grouped into three categories (See Table 1).

**TABLE 1:** Non-exhaustive list of red-teaming cases for different applications

Type of application	References	Summary	Key findings
Evasion & safeguard failure	Wittmann et al. (2025)	Tested biosecurity screening software against AI-generated protein sequences (synthetic homologs)	AI-generated sequences evaded detection in all systems tested; patch later improved detection to 97%
	Beal et al. (2023)	Evaluated accuracy of BLAST in flagging sequences of concern	∼34% false positives; 12% misidentification from minor sequence changes
	Fan et al. (2025)	Developed SafeProtein protocol to red-team protein foundation models using adversarial inputs	Up to 70% success in bypassing safeguards; showed models could generate restricted outputs
Exploitation of system & infrastructure vulnerabilities	Ney et al. (2017)	Used custom DNA sequences to trigger remote code execution during sequencing	Revealed critical vulnerability allowing potential pathway for remote control of synthesis equipment
Misuse pathways & threat forecasting	Ingesson et al. (2025)	Explored future misuse scenarios (e.g., planted synthetic DNA evidence, targeted ethnic bioweapons)	Provided points of consideration for evaluating plausibility and impact of future scenarios
	Mouton et al. (2024)	Assessed role of internet and AI in enabling biological attacks	Highlighted accessibility risks and misuse pathways enabled by information access
	Elgabry and Camilleri (2022)	Combined red-teaming with delphi-style forecasting for biotech-enabled criminal risks	Structured anticipation of emerging criminogenic threats

### 3.1 Evasion and safeguard failure

Since at least 2004, US government agencies have suggested using red-teaming approaches to appraise and validate biodefence strategies (Zhang and Gronvall, 2020). This approach tests whether defensive systems such as screening tools and AI safeguards would hold up against adversarial sequence manipulation or prompt-based attacks. Recently, Wittmann et al. carried out a red-teaming exercise on select biosecurity screening software. The exercise evaluated whether these systems were equipped to flag ordered sequences that were generated by protein sequence generative models. These models can suggest changes to amino acid sequences of a protein while preserving protein function, which means bad actors can use such tools to create hazardous proteins but escape detection by the use of these “synthetic homologs”. Alarmingly, AI-generated variants of certain protein sequences evaded detection across at least one biological sequence screening system in all cases. The group then added a patch to address this vulnerability, which successfully flagged 97% of such AI-formulated homologs (Wittmann et al., 2025). Another group examined the reliability of BLAST, the preeminent homology screening tool, in identifying sequences of concern relating to pathogens and toxins by screening it against NCBI’s protein database (Beal et al., 2023). An average of 34% of BLAST results incorrectly flagged similarity to sequences of concern from pathogens or toxins. 12% of sequences were misidentified because of the addition of only one amino acid. The authors declared that they chose not to publish cases where sequences of concern were missed by BLAST to avoid an information hazard.

Fan et al. has laid out a protocol called SafeProtein for systematic red-teaming of protein foundation models to limit biosafety risks in the context of masked sequences and structures. This effort was directly inspired by adversarial testing employed for LLMs, and the designed inputs were shown to bypass existing safety mechanisms. Up to 70% adversarial attempts succeeded for commonly available protein modelling tools, demonstrating that such models can be hijacked to design potentially harmful products (Fan et al., 2025). Similarly, Zhang et all has developed GeneBreaker, a framework to evaluate jailbreak vulnerabilities of DNA foundation models (Zhang et al., 2025). These efforts are supported by initiatives such as the Biosecurity Safeguards for Generative AI that is building capacity and knowledge to address AI-Biosecurity challenges (BioSafe GenAI. n.d.). Such attempts parallel the mainstream red-teaming employed in AI tools design to probe whether generative AI can be deployed to produce hazardous outputs (Wang et al., 2025).

### 3.2 Exploitation of system and infrastructure vulnerabilities

Red-teaming can also help probe whether vulnerabilities within existing benign tools can be potentially exploited by bad actors to weaponize the systems, infrastructure and platforms that support biological sciences research. Ney et al. used adversarial attacks via tailored DNA sequences that, when sequenced and processed, were successful in giving the attacker remote code execution. This serious security flaw could allow bad actors to remotely access and control components of important laboratory systems (Ney et al., 2017).

### 3.3 Misuse pathways and threat forecasting

The identification of misuse pathways and use of threat forecasting enables system-level probing of how technical capabilities might be combined into real-world threats. It is crucial to build early warning tools and identify future vulnerabilities before they materialise. For instance, Ingesson et al. forecasted the possibilities ranging from planting artificial DNA to frame individuals for designing bioweapons that target certain ethnicities. Such red-teaming-based feasibility analyses are essential for policymakers and officials plan contingency and mitigation measures (Ingesson et al., 2025). Mouton et al. conducted a large-scale red-teaming exercise to assess the potential of the internet and AI models in enabling biological attacks by providing relevant information (Mouton et al., 2024). Red-teaming, in conjunction with Delphi-style anticipation activities, has been used to assess criminogenic potential of biotech advancements (Elgabry and Camilleri, 2021; Elgabry et al., 2022).

## 4 Discussion

The limitations of current SNAT biosecurity mechanisms: screening-level constraints, system-level issues and increasing adaptive pressure from emerging sectors, can be directly mapped to red-teaming capabilities. Red-teaming offers security benefits that other assessment mechanisms such as certifications do not. Red-teaming can holistically evaluate SNAT system components across technical, procedural and human layers and provide realistic, adversary simulation testing that reveals hidden vulnerabilities. The systems-level overview can provide unparalleled insights, given that risks often emerge due to the interface of bad actors, vulnerable technologies and governance lapses. For instance, red-teaming can simultaneously probe customer vetting (background checks, misrepresentation) to screening pipelines (sensitivity to detect masking, use of AI design tools) to supply chain (order fragmentation, cross-jurisdiction orders) to downstream misuse (redistribution, secondary use of concern).

As a result, red-teaming also enables anticipatory identification of novel threats and allows corrective actions to address regulatory lags and close loopholes in frameworks promptly before they are exploited. The proposed regulatory interventions can themselves be tested by red-teaming to assuage robustness and scalability concerns.

### 4.1 Ethics and safety considerations

Integration of red-teaming into the SNAT biosecurity toolkit requires careful consideration of its inherent risks. These challenges range from technical constraints to ethical concerns. A primary concern is that of information hazard from dual-use knowledge. At its core, red-teaming exposes vulnerabilities, tools that can be misused and protocols that confound safeguards. Such findings, if handled recklessly, can nullify barriers and create possibilities for malicious users (Wittmann et al., 2025).

Therefore, a balance must be struck to securely share vulnerability assessments with trusted stakeholders through carefully built disclosure mechanisms for handling sensitive information (Esvelt, 2018; Lewis et al., 2019). Vulnerability disclosure and coordinated disclosure frameworks provide mechanisms for safe and confidential distribution of information across software providers and avoids duplication of efforts (Householder et al., 2017). Vendors must also keep customer and sequence data confidential to maintain trust. They may risk loss of market share if customers have concerns regarding the sharing of proprietary material (Maurer et al., 2009).

Further, red-teaming in biological sciences lacks standardisation. There may be discrepancies between defining metrics and the composition of realistic adversarial scenarios, as well as what constitutes an appropriate redressal. There is little consensus on what may be deemed “safe” levels of red-team testing (Carter et al., 2023). There might also be concerns surrounding the dual-use potential of such exercises themselves and their conflict with commonly applied principles of research ethics that typically forbid intentionally hazardous testing and experimentation (Carter et al., 2023).

Therefore, low-risk, but safe red-teaming exercises may miss critical issues, while a stricter one may create legal and ethical controversies, jeopardising progress in the field. Incorporation of benign proxies, simulated environments and formal legal and ethical review, such as the involvement of law enforcement and government agencies (Esvelt, 2024), can minimise risks and still preserve the diagnostic value of red-teaming.

### 4.2 A cross-disciplinary challenge

The adaptive pressures on SNAT increasingly require cross-disciplinary integration across synthetic biology, artificial intelligence, cybersecurity and policy. AI developers may lack the expertise to evaluate the biological consequences of their tools, and life science researchers may not be trained to anticipate AI misuse or adversarial risk. The latter was illustrated when Urbina et al. demonstrated how openly available drug discovery software can be realigned to produce chemical weapons, an unintended outcome never envisioned by developers or users (Urbina et al., 2022). New interactions between biotechnology and cyber technologies require cross-domain risk evaluation and showcase how tools safe-to-use in one domain can be repurposed to create vulnerabilities in another domains (Peccoud et al., 2018).

### 4.3 Hybrid governance

In the present set-up, private sector gatekeeping is in effect the primary biosecurity control on SNAT. While few commercial DNA synthesis vendors are known to conduct in-house red-teaming, the uniformity, scale and results of such exercises are not known (Kane and Parker, 2024). Given that a significant part of the actual implementation of screening guidelines, such as the IGSC Harmonised Protocol, is left to individual companies, and uneven adoption across regions and vendors is a known fact, governments must play a proactive role in supporting and standardising red-teaming practices. This can also encompass funding external assessments and establishing standardised benchmarks (Carter et al., 2023).

The community governance model led by IGSC can be leveraged to develop community-driven norms. Adding regulatory layers, such as red-teaming, demands investment of resources by DNA synthesis providers. In an industry where technological advancements are bringing costs down, this can create economic and competitive burdens (Beal and Alexanian, 2025). Therefore, it is important to incentivise screening and ensure that incorporating red-teaming does not make companies that adopt it less competitive. Involvement of industry partners, industry-wide red-teaming, and incentivisation is thus essential to ensure that red-teaming practices are scalable, sustainable, structured and uniform across the SNAT ecosystem.

Any red-teaming attempt will be incomplete without consequent coordinated response mechanisms. Red-teaming in SNAT must be conceptualised as part of an adaptive governance model that evaluates, provides feedback and offers mitigation measures in a continuous loop (Evans et al., 2020). Responsible disclosure frameworks inspired by initiatives like the CERT CVD can be adapted for sharing red-teaming findings among synthetic nucleic acid providers (Householder et al., 2017). This establishes the core necessity of making SNAT governance dynamic, continuous and capable of keeping up or rather outrunning, the technological advancements that enable creative misuse. Thus, institutionalising red-teaming into current oversight mechanisms will require sustained cross-sectoral coordination among academia, industry, security agencies and policymakers.

### 4.4 Changing the culture of bio-responsibility

The participation of stakeholders is essential to the success of any governance mechanism. Examples have shown that researchers and users are often not trained or even aware of dual-use risks or potential misuse possibilities (Urbina et al., 2022). This naivety limits the effectiveness of any red-teaming initiative and demands that any red-teaming embedding must include targeted awareness and training programmes to seed biosecurity, ethics and safe-use considerations into everyday practice (Peccoud et al., 2018). Such efforts should encourage transparency and create clear pathways for secure reporting of potential risks and biosecurity breaches in a competitive industry where reputational risk, economic burden and legal issues can discourage disclosures that reveal weaknesses in a company’s proprietary systems (Maurer et al., 2009; Urbina et al., 2022).

## 5 Conclusion

SNAT biosecurity must add anticipatory approaches to its traditional reactive control-guided static outlook to keep up with the rapid evolution of SNAT as well as developments in adjacent fields like AI. The reliance on known threats, uneven enforcement and limited pre-emptive capacity have made the SNAT biosecurity vulnerable to adaptive risks.

In this context, red-teaming offers solutions that can strengthen current approaches. Systematic probing for weaknesses in screening systems, vetting pipelines and governance structures can help identify emerging risks. However, as we point out, its implementation requires careful evaluation and mitigation of its own dual-use challenges. Any embedding of red-teaming must involve robust institutional and policy frameworks that encompass defined ‘safety’ mandates, standardisation, coordinated oversight and responsible information sharing. It is equally important to create awareness, establish secure reporting and information sharing structures, and implement appropriate safeguards to regulate this exercise.
